# Beyond the Nodes: Atypical Presentations of Non-Hodgkin's Lymphoma Across Organs

**DOI:** 10.7759/cureus.107698

**Published:** 2026-04-25

**Authors:** Swetha A, L D'Cruze, B Archana, Sandhya Sundaram, Nagarajan Priyathersini

**Affiliations:** 1 Department of Pathology, Sri Ramachandra Institute of Higher Education and Research, Chennai, IND; 2 Department of Pathology and Laboratory Medicine, Sri Ramachandra Institute of Higher Education and Research, Chennai, IND

**Keywords:** atypical presentation, burkitt lymphoma, diffuse large b-cell lymphoma, extranodal lymphoma, immunohistochemistry, non-hodgkin's lymphoma

## Abstract

Introduction

Extranodal presentation of non-Hodgkin's lymphoma (NHL) poses a diagnostic challenge due to its diverse clinical, morphological, and pathological features. These lymphomas arise in organs other than lymph nodes. Immunohistochemistry (IHC) plays a crucial role in the confirmation and subtyping of these lesions.

Objective

The aim of this study was to identify different patterns of extranodal involvement in NHL and to list the common sites of involvement.

Materials and methods

This retrospective case series included eight cases of atypical extranodal presentations of NHL across organs diagnosed by the Department of Pathology of Sri Ramachandra Institute of Higher Education and Research, Chennai, India, between 2019 and 2025.

Results

The cases involved extranodal lymphomas of the cervix (Burkitt's lymphoma), stomach (B-cell lymphoma), rectum (diffuse large B-cell lymphoma (DLBCL)), paratesticular mass (B-cell lymphoma), ileum (T-cell lymphoma), mediastinum (DLBCL), soft tissue of the thumb (mantle cell lymphoma), and breast (B-cell lymphoma). Each case demonstrated distinct clinicopathological features, emphasizing the need for different diagnoses and treatment approaches.

Conclusion

This case series highlights eight cases of NHL from various organs. Awareness of such atypical presentations is essential for early diagnosis and management.

## Introduction

Non-Hodgkin's lymphoma (NHL) represents a heterogeneous group of lymphoid malignancies characterized by diverse clinical, morphological, immunophenotypic, and genetic features. Globally, NHL accounts for approximately 4% of all malignancies and remains one of the most common hematologic cancers [1,2]. These neoplasms arise from the clonal proliferation of lymphoid cells, including B lymphocytes, T lymphocytes, and natural killer (NK) cells at different stages of differentiation, resulting in a wide spectrum of biological behaviors and clinical presentations. Among these, B-cell lymphomas constitute nearly 85-90% of cases, whereas T-cell and NK-cell lymphomas account for a smaller proportion [2].

The current classification of NHL is based on the World Health Organization (WHO) classification, which integrates morphological, immunophenotypic, genetic, and clinical features. Broadly, NHL is categorized into B-cell and T-cell/NK-cell neoplasms. B-cell lymphomas include entities such as diffuse large B-cell lymphoma (DLBCL), follicular lymphoma, mantle cell lymphoma, and marginal zone lymphoma, whereas T-cell and NK-cell lymphomas comprise peripheral T-cell lymphoma, anaplastic large cell lymphoma, and extranodal NK-cell/T-cell lymphoma. These entities are further stratified into indolent and aggressive subtypes based on clinical behavior, with important implications for prognosis and management.

Traditionally, NHL predominantly involves lymph nodes and other lymphoid organs such as the spleen and bone marrow. However, extranodal involvement is increasingly recognized and has been reported in approximately 30-40% of NHL cases, with even higher frequencies in certain geographic regions, particularly Asia [3]. Extranodal lymphomas may arise in virtually any organ system but most commonly affect the gastrointestinal (GI) tract, skin, central nervous system (CNS), and testes.

In rare instances, NHL may occur in unusual anatomical locations that are not typically associated with lymphoid tissue, including the cervix, breast, thyroid gland, and various soft tissues. Such atypical presentations can closely mimic primary malignancies of the involved organs, posing significant diagnostic challenges for clinicians, radiologists, and pathologists. Early recognition of these uncommon manifestations is essential for accurate diagnosis and appropriate management [4].

Despite increasing recognition of extranodal NHL, the literature predominantly focuses on common sites such as the GI tract and skin. Reports describing involvement of rare and unusual anatomical locations remain limited, particularly in the form of well-characterized case series. Moreover, these atypical presentations are often under-recognized and may lead to misdiagnosis due to their close resemblance to primary malignancies of the affected organs.

In this study, we present a case series highlighting atypical extranodal presentations of NHL involving uncommon organ systems. This series aims to emphasize the diagnostic challenges encountered in routine pathology practice, underscore the importance of maintaining a high index of suspicion for lymphoma in unusual extranodal lesions, and ultimately aid in preventing diagnostic delays or mismanagement. By documenting these rare presentations, this study contributes to the existing literature and provides practical insights for pathologists and clinicians in daily practice.

## Materials and methods

Study design

This retrospective case series included eight cases of atypical presentations of NHL involving extranodal organs diagnosed in the Department of Pathology of Sri Ramachandra Institute of Higher Education and Research, Chennai, India, over a six-year period from 2019 to 2025. All consecutive cases fulfilling the inclusion criteria during the study period were included to minimize selection bias.

Inclusion and exclusion criteria

Cases included in the study were those diagnosed as primary extranodal NHL with no evidence of primary nodal involvement at the time of diagnosis. The designation of primary extranodal lymphoma was established based on clinical, radiological, and pathological correlation. The definition was applied in accordance with established criteria in the literature, wherein the predominant disease burden was extranodal with absent or minimal nodal involvement.

Staging workup was performed using available clinical and radiological data, including contrast-enhanced computed tomography (CECT) and/or positron emission tomography-computed tomography (PET-CT), to assess nodal and extranodal disease distribution. Bone marrow examination findings, wherever available, were also reviewed to exclude systemic involvement. Variations in staging completeness due to the retrospective nature of the study were acknowledged.

Only cases with predominant extranodal disease and no evidence of widespread nodal involvement at presentation were included. Cases with secondary extranodal involvement or those presenting as primary nodal lymphomas were excluded.

Data collection

Patients' demographic and clinical details were obtained from the hospital information system. Relevant clinical records, imaging findings, and laboratory data were reviewed. Histopathological slides stained with hematoxylin and eosin (H&E) were examined, and immunohistochemical (IHC) studies performed for diagnostic confirmation were reviewed along with the corresponding pathology reports.

Histopathological and immunohistochemical evaluation

All available histopathological slides were reassessed to evaluate morphological features, including tumor architecture, cytological characteristics, pattern of infiltration, presence of necrosis, mitotic activity, and associated stromal or inflammatory response.

IHC analysis for lymphoma subtyping included a panel of B-cell markers (e.g., CD20, CD79a), T-cell markers (e.g., CD3), and additional markers such as CD5, CD10, BCL2, BCL6, cyclin D1, and Ki-67, wherever available. IHC interpretation was performed using standard diagnostic thresholds as per institutional protocols. These findings were used to confirm the diagnosis and classification of lymphoma.

Statistical analysis

As this study represents a descriptive case series with a limited number of cases, descriptive analysis was performed, including calculation of frequencies, percentages, and simple tabulation to summarize the clinical, pathological, and IHC findings.

Ethical approval

The study was conducted following approval from the Institutional Research Ethics Committee of Sri Ramachandra Institute of Higher Education and Research (approval number: CSP-MED/25/AUG/120/234). The study was carried out in accordance with institutional ethical standards.

## Results

Case 1

Clinical Presentation

A 26-year-old woman presented with vomiting and abdominal pain for five days.

Clinical Details

No significant past medical history was noted. Routine laboratory investigations were within normal limits.

Imaging Findings

Imaging revealed a bulky uterus with a hypoechoic cervical lesion showing posterior wall infiltration into the left parametrium.

Histopathology

Microscopic examination showed diffuse sheets of atypical lymphoid cells with high mitotic activity.

IHC

Tumor cells were positive for CD45, CD20, c-Myc, MuM1, Bcl6, and CD10 and negative for PanCK, Bcl2, and cyclin D1. Ki-67 labeling index was approximately 100%. Background T cells were highlighted by CD3 and CD5. 

Primary Malignancy Details

The following were the details of the primary malignancy: location: cervix; type: high-grade B-cell lymphoma; subtype: Burkitt's lymphoma; extranodal involvement: yes (uterine cervix); and nature: hematolymphoid malignancy.

Final Diagnosis

**Figure 1:**
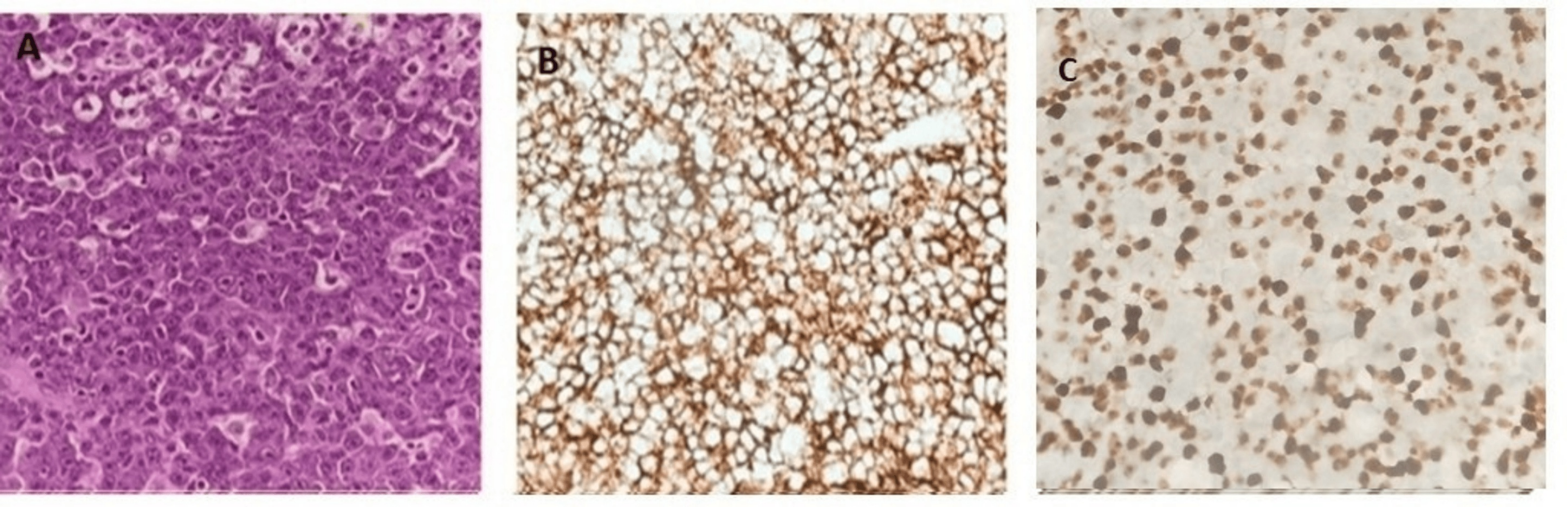
Histological and IHC findings of Burkitt's lymphoma: cervix (A) Diffuse sheets of atypical lymphoid cells in the cervix on H&E stain (40× magnification). (B) Tumor cells showing strong membranous positivity for CD45 on IHC (40× magnification). (C) Ki-67 immunostaining demonstrating the index of approximately 100% (40× magnification). IHC: immunohistochemistry; H&E: hematoxylin and eosin

The patient was diagnosed with Burkitt's lymphoma of the cervix (Figure 1).

Management

The patient was planned for chemotherapy (intensive Burkitt's lymphoma protocol).

Case 2

Clinical Presentation

A 53-year-old woman presented with disease relapse.

Clinical Details

The patient had received two cycles of rituximab, gemcitabine, and oxaliplatin (R-GEM-OX) chemotherapy prior to presentation.

Imaging Findings

Magnetic resonance imaging (MRI) revealed hyperintense circumferential thickening involving the anal canal and lower rectum. A positron emission tomography (PET) scan showed fluorodeoxyglucose (FDG)-avid circumferential wall thickening (maximum thickness 7 mm) in the same region.

Histopathology

Examination of the colonic mucosa showed infiltration by atypical lymphoid cells arranged in sheets.

IHC

Tumor cells were positive for CD45 and CD20 and negative for CD3, CD56, synaptophysin, and PanCK. The Ki-67 labeling index was approximately 80%.

Primary Malignancy Details

The following were the details of the primary malignancy: location: rectum and anal canal; type: B-cell NHL; subtype: DLBCL, germinal center B-cell (GCB) type; disease status: relapsed lymphoma; and nature: hematolymphoid malignancy.

Final Diagnosis

**Figure 2:**
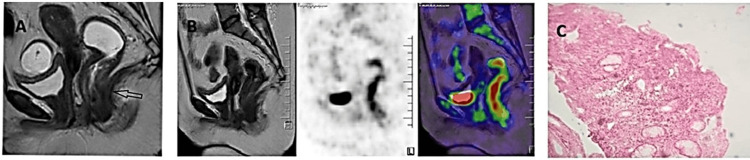
Radiological and histological findings of DLBCL: rectum (A) MRI showing hyperintense circumferential thickening involving the anal canal and lower rectum. (B) PET scan revealing reduction in metabolic activity and FDG-avid circumferential wall thickening involving the anal canal and lower rectum. (C) H&E showing diffuse sheets of atypical lymphoid cells in the rectum (40× magnification). DLBCL: diffuse large B-cell lymphoma; MRI: magnetic resonance imaging; PET: positron emission tomography; H&E: hematoxylin and eosin; FDG: fluorodeoxyglucose

The patient was diagnosed with relapsed DLBCL (GCB type) involving the rectum (Figure 2).

Management

The patient was continued on salvage chemotherapy (e.g., R-GEM-OX regimen).

Case 3 

Clinical Presentation

A 58-year-old man presented with bilateral lower limb weakness and loss of sensation for five days, with a history of a slip and fall one month prior. He was diagnosed with D2 metastatic disease with thoracic myelopathy.

Clinical Details

No significant past medical history was noted. Routine laboratory investigations were within normal limits.

Imaging Findings

PET scan revealed an FDG-avid lesion in the right inguinoscrotal region.

Histopathology

Biopsy from the paratesticular mass showed sheets of atypical lymphoid cells.

IHC

Tumor cells were positive for CD45 and CD20 and negative for cytokeratin and SALL4. CD3 highlighted background T lymphocytes.

Primary Malignancy Details

The following were the details of the primary malignancy: location: paratesticular region; type: B-cell NHL; subtype: DLBCL; extranodal involvement: yes; and nature: hematolymphoid malignancy.

Final Diagnosis

**Figure 3:**
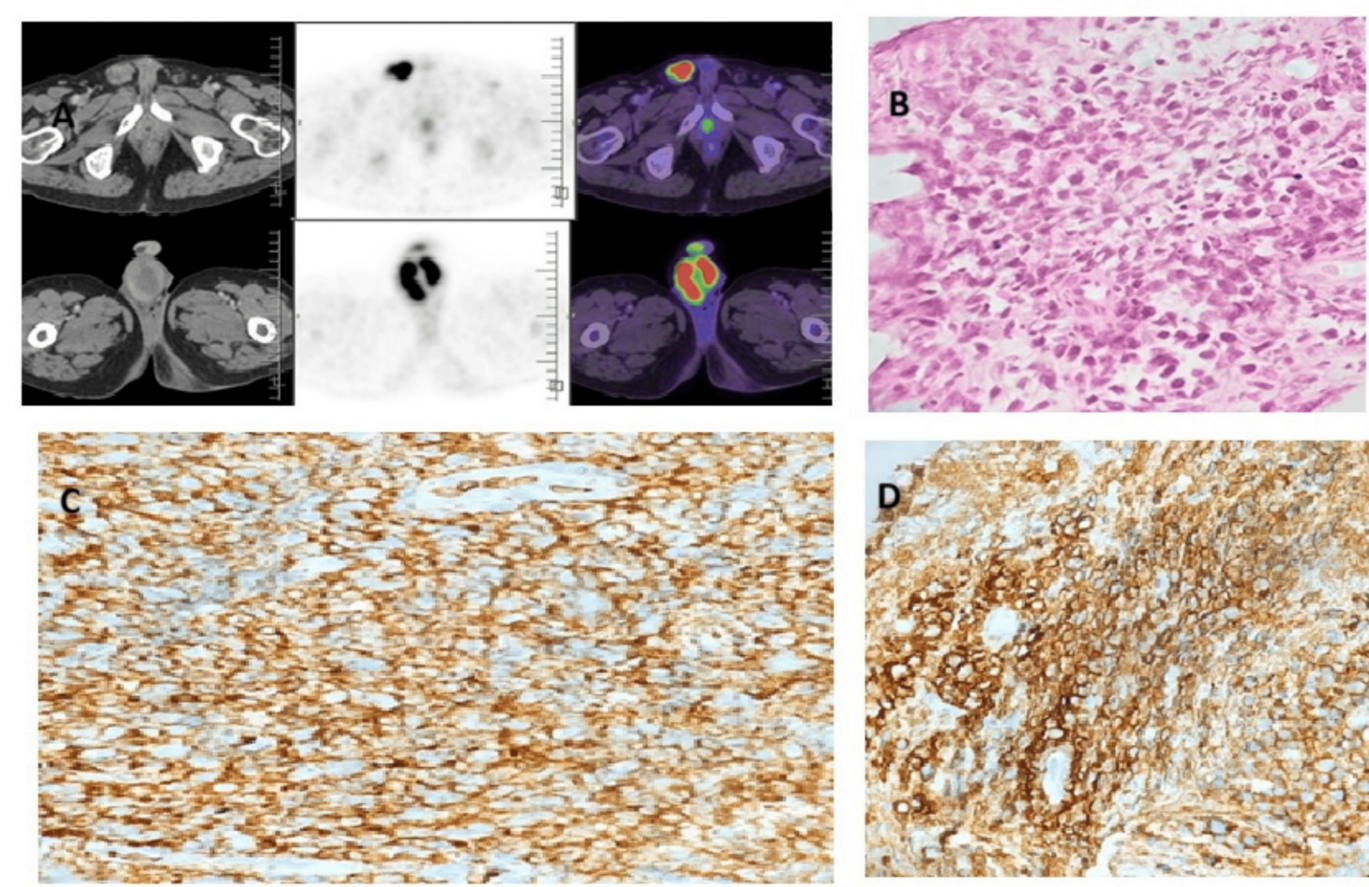
Radiological, histological, and IHC findings of DLBCL: paratesticular mass (A) PET scan revealing an FDG-avid lesion in the right inguinal scrotal mass. (B) H&E showing diffuse sheets of atypical lymphoid cells in the paratesticular mass (40× magnification). (C) Tumor cells positive for IHC CD45 (40× magnification). (D) Tumor cells positive for IHC CD20 (40× magnification). IHC: immunohistochemistry; DLBCL: diffuse large B-cell lymphoma; PET: positron emission tomography; H&E: hematoxylin and eosin; FDG: fluorodeoxyglucose

The patient was diagnosed with DLBCL involving the paratesticular region (Figure 3).

Management

The patient was planned for systemic chemotherapy (e.g., rituximab, cyclophosphamide, doxorubicin, vincristine, and prednisone (R-CHOP) regimen).

Case 4

Clinical Presentation

A 64-year-old man presented with abdominal pain and difficulty passing stools for two weeks.

Clinical Details

No significant past medical history was noted.

Imaging Findings

Imaging revealed posterior wall thickening of the ileum.

Histopathology

Biopsy from the ileum showed dense infiltration by atypical lymphoid cells.

IHC

Tumor cells were positive for CD3, CD45, and c-Myc and negative for CD20, Bcl2, Bcl6, CD10, MuM1, cyclin D1, and cytokeratin. The Ki-67 labeling index was approximately 90%.

Primary Malignancy Details

The following were the details of the primary malignancy: location: ileum; type: NHL; subtype: likely T-cell lymphoma; and nature: hematolymphoid malignancy.

Final Diagnosis

**Figure 4:**
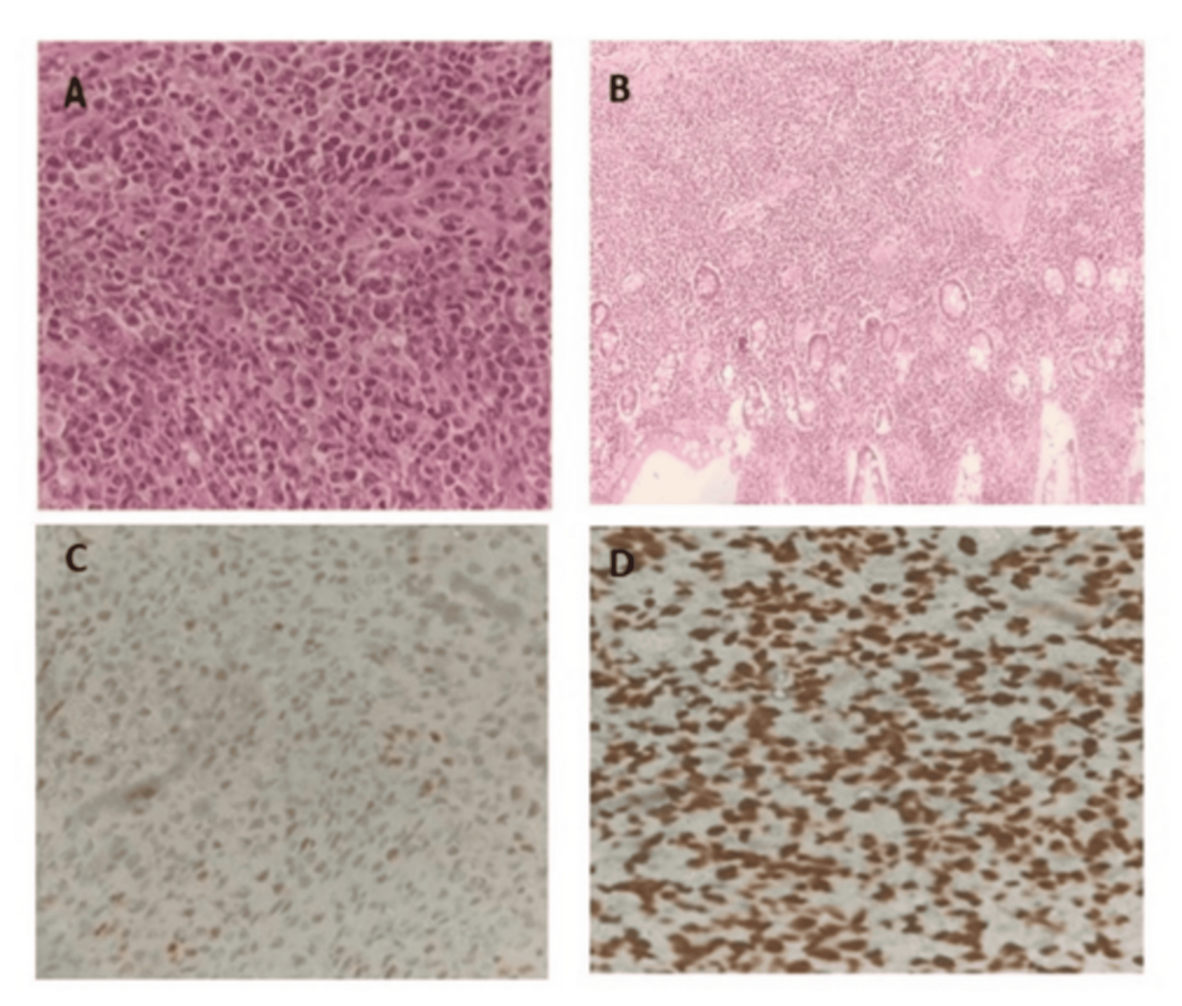
Histological and IHC findings of NHL of T-cell lineage: ileum (A) H&E showing dense sheets of atypical lymphoid cells (40× magnification). (B) H&E showing atypical lymphoid cells along with adjacent ileal glands (20× magnification). (C) Tumor cells positive for IHC c-Myc (40× magnification). (D) Ki-67 proliferation index: 90% (40× magnification). IHC: immunohistochemistry; NHL: non-Hodgkin's lymphoma; H&E: hematoxylin and eosin

The patient was diagnosed with NHL of probable T-cell lineage involving the ileum (Figure 4).

Management

The patient was advised to undergo further hematological evaluation and chemotherapy.

Case 5

Clinical Presentation

A 28-year-old woman presented with breathlessness and was admitted for the initiation of chemotherapy.

Clinical Details

No significant past medical history was noted.

Imaging Findings

PET-CT showed a large ill-defined mass lesion in the anterior mediastinum extending into the pretracheal region.

Histopathology

Biopsy from the mediastinal mass showed sheets of large atypical lymphoid cells.

IHC

Tumor cells were positive for CD20, CD10, and Bcl6 and negative for CD3, PanCK, SALL4, CD5, Bcl2, MuM1, c-Myc, and cyclin D1. The Ki-67 labeling index was approximately 90%.

Primary Malignancy Details

The following were the details of the primary malignancy: location: anterior mediastinum; type: B-cell NHL; subtype: DLBCL (GCB type); and nature: hematolymphoid malignancy.

Final Diagnosis

**Figure 5:**
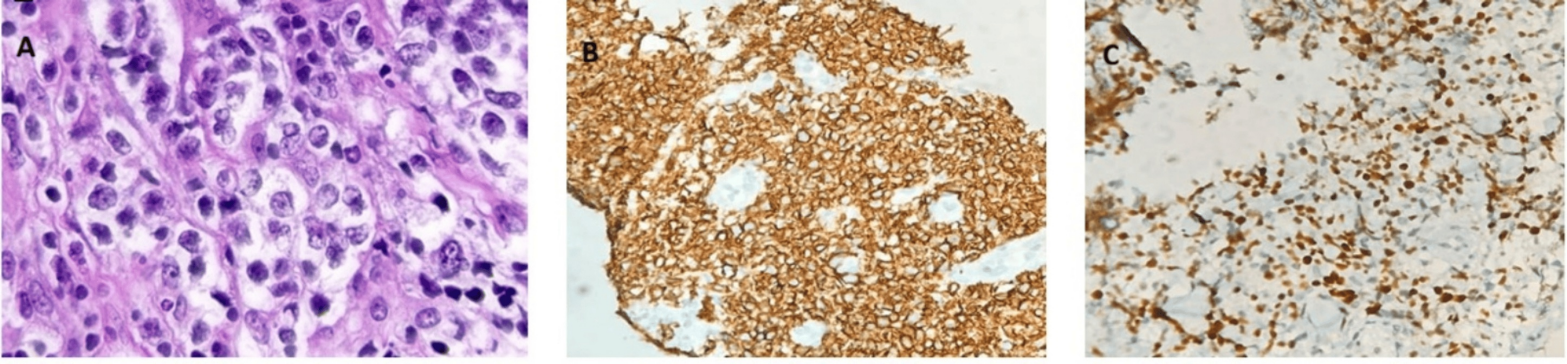
Histological and IHC findings of DLBCL: mediastinum (A) Histopathological features from mediastinal mass showing large atypical lymphoid cells arranged in sheets (40× magnification). (B) Tumor cells positive for IHC CD10 (40× magnification). (C) Tumor cells positive for IHC Bcl6 (40× magnification). IHC: immunohistochemistry; DLBCL: diffuse large B-cell lymphoma

The patient was diagnosed with DLBCL (GCB type) of the mediastinum (Figure 5).

Management

The patient was started on R-CHOP chemotherapy.

Case 6 

Clinical Presentation

A 65-year-old man presented with a soft tissue swelling involving the thumb.

Clinical Details

No significant past medical history was noted.

Imaging Findings

MRI revealed a soft tissue intensity lesion in the subcutaneous plane overlying the dorsal and lateral aspects of the first metacarpal extending to the wrist joint.

Histopathology

Examination showed infiltrating sheets of atypical lymphoid cells.

IHC

Tumor cells were positive for CD20 and cyclin D1.

Primary Malignancy Details

The following were the details of the primary malignancy: location: soft tissue of the thumb; type: B-cell NHL; subtype: mantle cell lymphoma; extranodal involvement: yes (rare site); and nature: hematolymphoid malignancy.

Final Diagnosis

**Figure 6:**
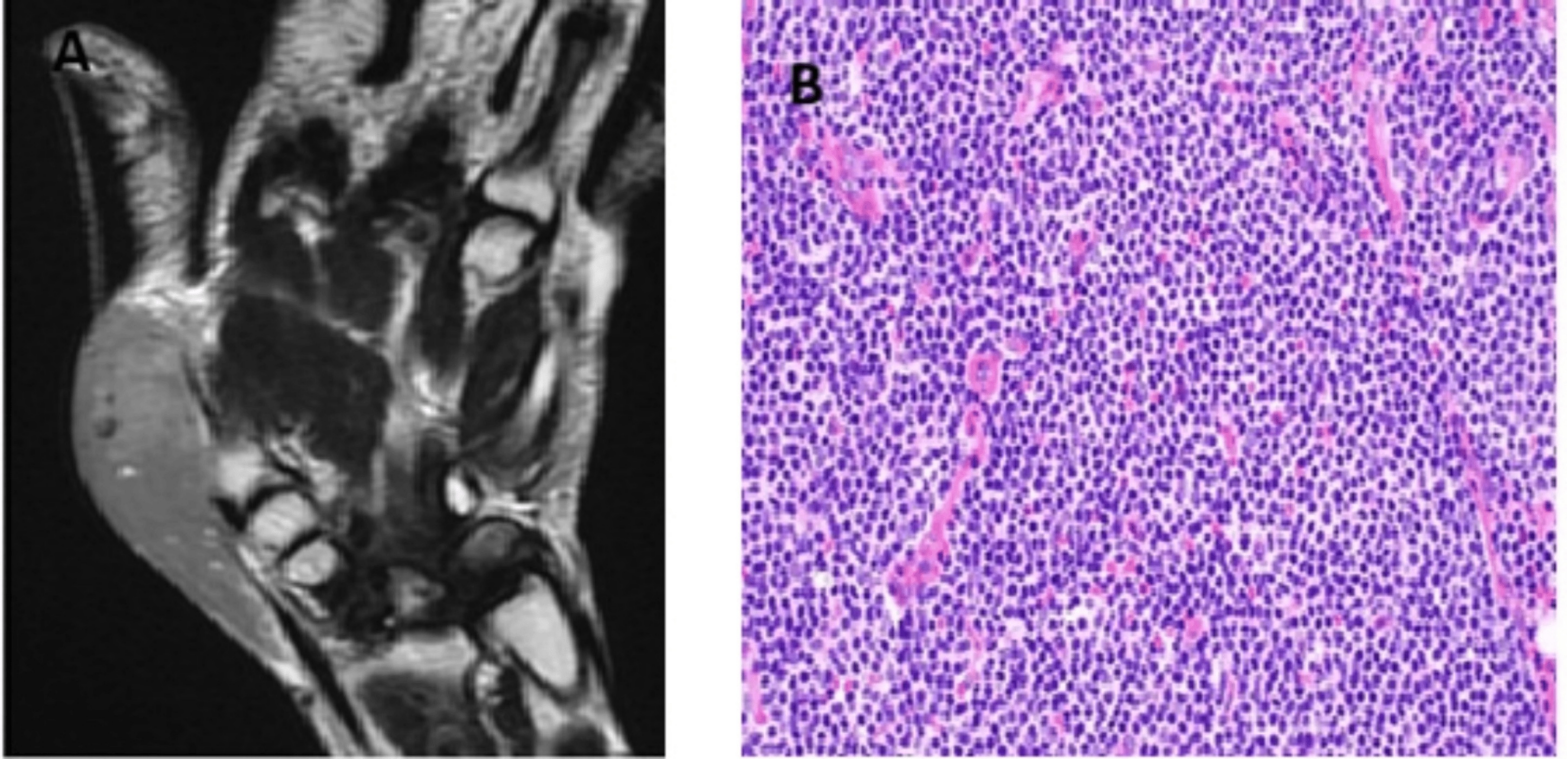
Radiological and histochemical findings of mantle cell lymphoma: thumb (A) MRI showing a soft tissue intensity lesion within the subcutaneous plane overlying the dorsal and lateral aspects of the first metacarpal and wrist joint. (B) H&E showing infiltrating tumor cells of atypical lymphoid cells arranged in sheets (40× magnification). MRI: magnetic resonance imaging; H&E: hematoxylin and eosin

The patient was diagnosed with mantle cell lymphoma involving the soft tissue of the thumb (Figure 6).

Management

The patient was planned for systemic chemotherapy and further staging workup.

Case 7

Clinical Presentation

A 60-year-old woman presented with severe abdominal pain and vomiting.

Clinical Details

No significant past medical history was noted.

Imaging Findings

MRI revealed asymmetric circumferential wall thickening of the stomach involving the greater curvature and extending along the lesser curvature.

Histopathology

Gastric biopsy showed atypical lymphoid cell infiltration.

IHC

Tumor cells were positive for CD20 and CD45 and negative for cytokeratin and CDX2.

Primary Malignancy Details

The following were the details of the primary malignancy: location: stomach; type: B-cell NHL; subtype: not further classified; and nature: hematolymphoid malignancy.

Final Diagnosis

**Figure 7:**
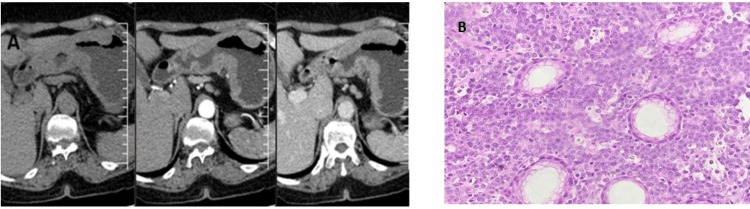
Radiological and histological features of B-cell lymphoma: stomach (A) MRI revealing asymmetric circumferential wall thickening in the stomach along the greater curvature and almost whole length along the lesser curvature. (B) Histopathological features from gastric biopsy revealing atypical lymphoid cells (40× magnification). MRI: magnetic resonance imaging

The patient was diagnosed with B-cell NHL of the stomach (Figure 7).

Management

The patient was advised chemotherapy following staging. Further subclassification was not performed as the available findings were sufficient to establish the diagnosis.

Case 8

Clinical Presentation

A 65-year-old woman with a known history of breast lymphoma presented with recurrent breast swelling.

Clinical Details

No significant past medical history was noted. Routine laboratory investigations were within normal limits.

Imaging Findings

These could not be retrieved due to the retrospective nature of the study.

Histopathology

Breast biopsy revealed atypical lymphoid cell infiltration.

IHC

Tumor cells were positive for CD20 and CD45.

Primary Malignancy Details

The following were the details of the primary malignancy: location: breast; type: B-cell NHL; disease status: recurrent lymphoma; and nature: hematolymphoid malignancy.

Final Diagnosis

**Figure 8:**
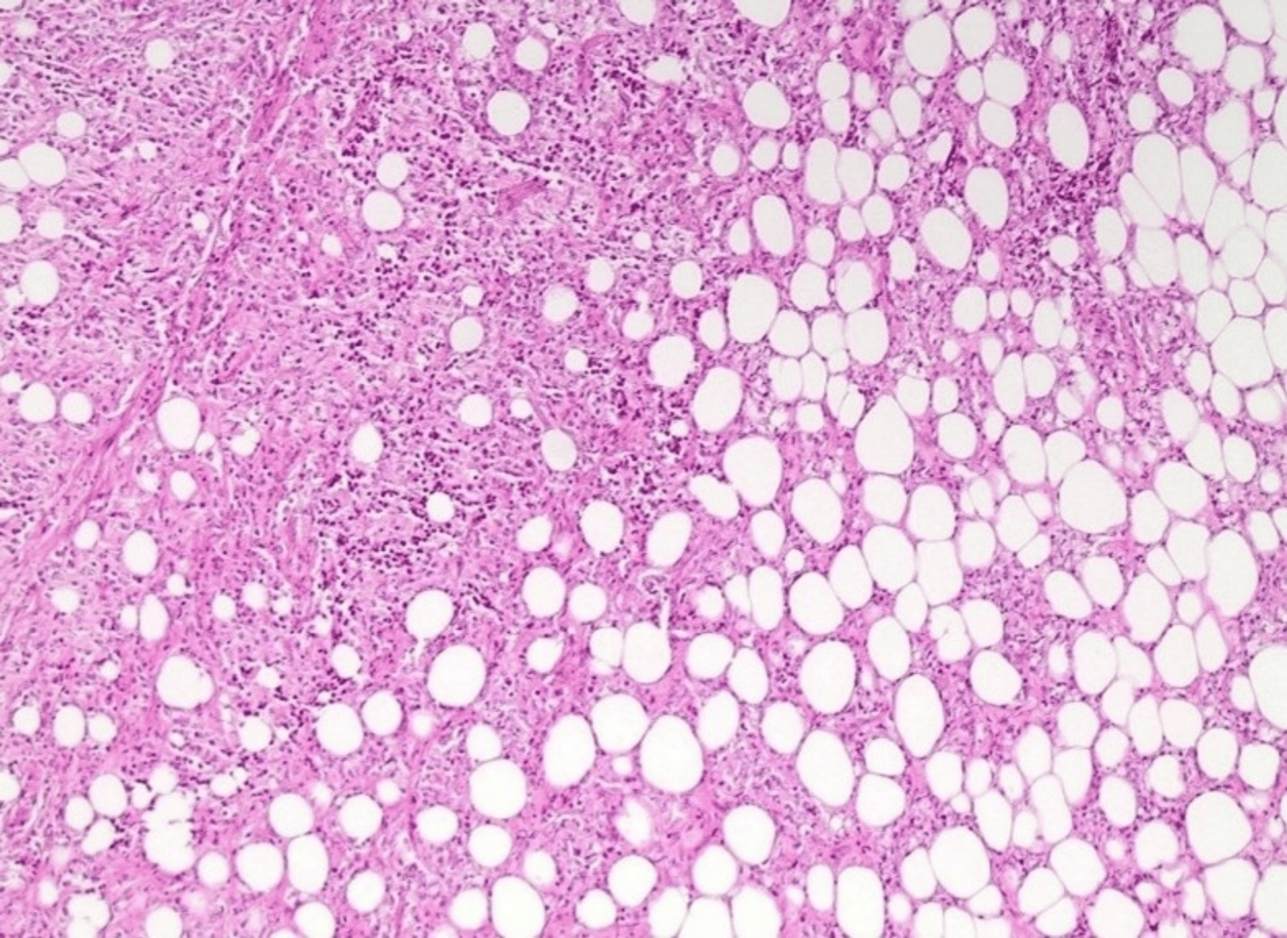
Histological features of B-cell lymphoma: breast

The patient was diagnosed with recurrent B-cell lymphoma of the breast (Figure 8).

Management

The patient was advised to undergo further chemotherapy.

A summary of clinical, radiological, and histopathological findings is provided in Table 1.

**Table 1:** Extranodal presentation of NHL NHL: non-Hodgkin's lymphoma; DLBCL: diffuse large B-cell lymphoma; MCL: mantle cell lymphoma; IHC: immunohistochemistry; MRI: magnetic resonance imaging; PET: positron emission tomography; FDG: fluorodeoxyglucose

Case	Age/sex	Site/organ	Clinical presentation	Imaging findings	IHC findings	Diagnosis
1	26/F	Cervix	Vomiting abdominal pain	MRI: bulky uterus with hypoechoic cervical lesion	CD45+, CD20+, c-Myc+, Bcl6+, MuM1+, CD10+, Ki-67 labeling index: 100%	B-cell NHL (Burkitt's lymphoma)
2	53/F	Rectum	Disease relapse	MRI: hyperintense circumferential thickening involving the anal canal and lower rectum	CD45+, CD20+, Ki-67 labeling index: 80-90%	B-cell NHL (DLBCL)
3	58/M	Paratesticular mass	Bilateral lower limb weakness	PET scan: FDG-avid lesion in the right inguinal scrotal mass	CD45+, CD20+	B-cell NHL (DLBCL)
4	64/M	Ileum	Difficulty in passing stools, abdominal pain	MRI: posterior wall ileal thickening	CD45+, c-Myc+	T-cell NHL
5	28/F	Mediastinum	Breathlessness	PET scan: large ill-defined malignant-looking mass lesion in the mediastinum	CD20+, CD10+, Bcl6+	B-cell NHL (DLBCL)
6	65/M	Thumb	Soft tissue swelling of the thumb	MRI: soft tissue intensity lesion within the subcutaneous plane overlying the dorsal and lateral aspects of the first metacarpal and wrist joint	CD20+, cyclin D1+	B-cell NHL (MCL)
7	60/F	Stomach	Abdominal pain, vomiting	MRI: asymmetric circumferential wall thickening in the stomach along the greater curvature and almost whole length along the lesser curvature	CD20+, CD45+	B-cell NHL of the stomach
8	65/F	Breast	Recurrence of breast swelling	-	CD20+, CD45+	B-cell NHL of the breast

## Discussion

NHLs comprise a heterogeneous group of lymphoid malignancies with considerable variability in clinical presentation, histopathology, immunophenotype, and prognosis [1]. Although nodal disease remains the most common manifestation, extranodal involvement is observed in approximately 30-40% of cases and may exceed 50% in certain regions, particularly in Asia [2]. The present case series highlights eight cases of primary extranodal NHL involving uncommon anatomical sites, emphasizing the diagnostic challenges and the pivotal role of histopathology and IHC in establishing an accurate diagnosis. These findings are in concordance with the current WHO classification of hematolymphoid tumors and previously published reviews, which emphasize the importance of integrating morphological, immunophenotypic, and molecular features for accurate diagnosis.

Lymphomas are classified according to the WHO classification of hematolymphoid neoplasms based on morphology, immunophenotype, and molecular features. DLBCL, the most common subtype, is further categorized into GCB and activated B-cell subtypes, which have prognostic significance. Current guidelines recommend IHC and PET-CT for diagnosis and staging, with R-CHOP-based regimens forming the mainstay of treatment. The cases in our series demonstrate varied extranodal presentations, which are consistent with previously reported literature, where DLBCL is identified as the most common subtype of extranodal lymphoma and demonstrates variable clinical presentations depending on the site of involvement [3].

Spectrum of extranodal involvement

The GI tract is the most frequent site of extranodal NHL, accounting for nearly half of primary extranodal lymphomas [5]. In the present series, gastric and rectal involvement by DLBCL and an ileal lymphoma were observed. GI lymphomas often present with nonspecific symptoms such as abdominal pain, vomiting, or intestinal obstruction, and radiological findings may mimic carcinoma or inflammatory conditions. Therefore, histopathological examination remains indispensable for definitive diagnosis.

The gastric lymphoma in our series demonstrated CD20 and CD45 positivity, confirming B-cell lineage, which is consistent with previously published Indian and international studies [3,6]. Testicular and paratesticular lymphomas are known for their aggressive clinical behavior and a high risk of CNS relapse, highlighting the importance of accurate diagnosis and appropriate staging [7]. The rectal DLBCL showed a high Ki-67 index of approximately 80%, correlating with its aggressive biological behavior and responsiveness to R-CHOP-based chemotherapy. In contrast, the ileal lymphoma was negative for B-cell markers and demonstrated c-Myc positivity on IHC. Intestinal lymphomas, particularly those of T-cell origin, are rare and aggressive and are frequently associated with complications such as intestinal perforation or obstruction [8].

Overall, the distribution and clinicopathological features observed in our series are comparable to previously published case series, which also report the GI tract as the most common extranodal site with predominance of B-cell lymphomas.

Gynecologic and genitourinary involvement

Primary lymphoma of the cervix is extremely rare, accounting for less than 1% of extranodal lymphomas and less than 0.5% of cervical malignancies [9]. Similar rarity has been highlighted in earlier case reports and small case series, underscoring the diagnostic challenge posed by such unusual presentations [9]. In our series, the case of cervical Burkitt's lymphoma presented as a bulky cervical lesion that was clinically suspected to be carcinoma. Burkitt's lymphoma is a highly aggressive B-cell neoplasm characterized by MYC gene rearrangement and an exceptionally high proliferation index, with Ki-67 approaching 100% [10,11]. Early recognition is crucial, as Burkitt's lymphoma demonstrates a dramatic response to intensive chemotherapy regimens when diagnosed promptly.

Paratesticular lymphoma represents another uncommon extranodal manifestation, accounting for approximately 1-2% of NHL cases [12,13]. These lymphomas predominantly affect older males and are most commonly of the DLBCL subtype. In our case, tumor cells showed positivity for CD45 and CD20, confirming B-cell lineage, while cytokeratin staining was negative, thereby excluding epithelial or germ cell tumors. Testicular and paratesticular lymphomas are known for their aggressive clinical behavior and a high risk of CNS relapse, highlighting the importance of accurate diagnosis and appropriate staging.

Breast and soft tissue involvement

Primary breast lymphoma (PBL) is rare, constituting less than 0.5% of all breast malignancies and approximately 1-2% of extranodal lymphomas [14]. Clinically and radiologically, PBL closely mimics carcinoma of the breast, often leading to diagnostic confusion. In the present series, the patient presented with recurrent breast swelling, and biopsy revealed B-cell NHL confirmed by CD20 and CD45 positivity. The absence of epithelial markers helped exclude carcinoma. Most primary PBLs are DLBCLs, and treatment outcomes have improved significantly with combined chemoimmunotherapy and radiotherapy.

Soft tissue involvement by NHL is uncommon and may be misdiagnosed as sarcoma or metastatic disease. The mantle cell lymphoma involving the thumb in our series represents a particularly rare presentation. Mantle cell lymphoma is characterized by the overexpression of cyclin D1 resulting from the t(11;14)(q13;q32) translocation [15]. While the lymph nodes, bone marrow, and GI tract are the usual sites of involvement, soft tissue presentations have rarely been reported. Despite advances in targeted therapies, mantle cell lymphoma remains an aggressive lymphoma with frequent relapses, emphasizing the importance of early and accurate diagnosis.

Mediastinal involvement

Primary mediastinal large B-cell lymphoma (PMBCL) is a distinct clinicopathological entity accounting for approximately 2-3% of NHL cases and predominantly affecting young females. The mediastinal lymphoma in our series demonstrated CD20, CD10, and BCL6 positivity, consistent with the GCB subtype. PMBCL typically presents as a rapidly enlarging anterior mediastinal mass associated with compressive symptoms such as breathlessness and superior vena cava obstruction. Advances in chemoimmunotherapy regimens, including R-CHOP and dose-adjusted etoposide, prednisolone, oncovin/vincristine, cyclophosphamide, doxorubicin, and rituximab (DA-EPOCH-R), have significantly improved long-term survival rates, which now exceed 80% [16].

Diagnostic role of IHC

IHC played a central role in diagnosis and subtyping in all cases in this series. CD45 confirmed lymphoid origin, while lineage-specific markers such as CD20 and CD3 facilitated differentiation between B-cell and T-cell lymphomas. The high Ki-67 indices observed in Burkitt's lymphoma and DLBCL reflected their aggressive biological nature [16]. Cyclin D1 expression confirmed mantle cell lymphoma in the soft tissue case. Additionally, negative staining for epithelial markers such as cytokeratin and epithelial membrane antigen (EMA) helped exclude carcinomas, particularly in lesions involving the cervix, breast, and GI tract. Thus, IHC remains indispensable in the evaluation of extranodal lesions, especially at unusual anatomical sites.

Therapeutic and prognostic implications

Treatment strategies for extranodal NHL are largely determined by histologic subtype, disease stage, and site of involvement. R-CHOP remains the standard treatment regimen for aggressive B-cell lymphomas and has significantly improved patient outcomes. These observations are consistent with existing literature, which demonstrates improved survival outcomes in aggressive B-cell lymphomas with the use of rituximab-based chemoimmunotherapy. Burkitt's lymphoma requires intensive multi-agent chemotherapy, whereas T-cell lymphomas often demonstrate a poorer response to conventional treatment regimens. Mantle cell lymphoma typically follows a relapsing course despite an initial response to therapy, often requiring newer targeted treatments such as Bruton tyrosine kinase inhibitors and chimeric antigen receptor (CAR)-T-cell therapy [8].

Extranodal involvement may also have important prognostic implications. Testicular and breast lymphomas are associated with a higher risk of CNS relapse and may require prophylactic CNS therapy [5]. Gastric lymphomas generally demonstrate favorable outcomes when treated appropriately, whereas intestinal T-cell lymphomas are associated with poorer prognosis due to their aggressive behavior and frequent complications [9].

Strengths and limitations

The study has certain limitations. The retrospective design introduces inherent biases, including dependence on available clinical records and potential variability in staging workup across cases. Follow-up data were not consistently available, precluding the assessment of treatment response, survival outcomes, and prognostic correlations. The relatively small sample size, inherent to a case series, limits the generalizability of the findings. Additionally, molecular characterization and cytogenetic studies were not performed, which could have provided further insights into the biological behavior and subclassification of these lymphomas. Although IHC was performed for diagnostic confirmation, IHC images could not be retrieved for all cases, and only representative images have been included. Despite these limitations, this study underscores the importance of early biopsy, careful histopathological evaluation, and appropriate use of IHC in diagnosing extranodal lymphomas, particularly at unusual sites that may mimic other malignancies or inflammatory conditions.

## Conclusions

Extranodal presentations of NHL may occur in unusual anatomical sites and can mimic other malignancies or inflammatory conditions, thereby posing significant diagnostic challenges. Such atypical manifestations may lead to initial misdiagnosis, particularly when clinical and radiological findings suggest primary tumors of the involved organs.

The cases presented in this series highlight the diverse spectrum of extranodal involvement and underscore the importance of maintaining a high index of suspicion for lymphoma in unusual organ-based lesions. Careful histopathological examination supported by appropriate IHC markers plays a crucial role in establishing an accurate diagnosis.

Early recognition of these atypical presentations may facilitate timely diagnosis and appropriate patient management. A multidisciplinary approach involving clinicians, radiologists, and pathologists is essential for accurate diagnosis and optimal management.

However, given the small sample size and retrospective nature of this study, the findings should be interpreted with caution. This case series is exploratory in nature and aims to highlight rare presentations and diagnostic challenges rather than draw definitive conclusions. Further studies with larger cohorts, comprehensive molecular characterization, and detailed follow-up are warranted to better understand the clinical behavior, treatment response, and long-term outcomes of such rare extranodal presentations.
